# Diabetic Foot Risk Assessment among Patients with Type 2 Diabetes in Kenya

**DOI:** 10.24248/eahrj.v6i2.698

**Published:** 2022-11-30

**Authors:** James Ngoyo Nduati, Samwel Maina Gatimu, Yeri Kombe

**Affiliations:** a School of Public Health, Jomo Kenyatta University of Science and Technology, Nairobi, Kenya; b Diabetic Foot Foundation of Kenya, Nairobi, Kenya; c Kenya Medical Research Institute, Nairobi, Kenya

## Abstract

**Background:**

Screening for diabetic foot complications is often neglected, especially during routine and/or annual diabetes check-ups. We assessed the risk of diabetic foot complications among patients with type 2 diabetes in Kenya using the International Working Group on Diabetic Foot risk stratification guidelines to highlight the need for improved foot care.

**Methods:**

We conducted a descriptive cross-sectional study in Mathari National Teaching and Referral Hospital in Kenya between July and October 2015. Seven hundred patients with type 2 diabetes were identified and 147 were systematically sampled. A trained podiatrist examined patients, and urine and blood samples were taken for biochemical tests and assessed by the investigating team.

**Results:**

In total, 44(29.9%) men and 103(70.1%) women were sampled; 75(51.0%) were aged over 55 years, 113(76.9%) were overweight/obese, 117(79.6%) had poor glycaemic control and 125(85%) had never had their feet screened for complications. Thirty participants (20.4%) were categorised as being at high risk for developing diabetic foot complications while 54(36.7%) had moderate risk, 53(36.1%) had low risk and 10(6.8%) had no risk. Compared to other risk groups, those with moderate risk for developing diabetic foot problems had higher mean levels of glycated haemoglobin (9.4%), albumin-creatinine ratio (50.3) and high-density lipoprotein cholesterol (1.4 mmol/L) at presentation. No other differences in clinical and laboratory profiles were noted.

**Conclusion:**

Our results show high rates of obesity, and poor glycaemic control in patients with type 2 diabetes and 56.5% of patients are categorised as being a moderate-to-high risk for foot problems. This highlights the need for healthcare professionals and patients in Kenya to be sensitised regarding the importance of foot screening to prevent lower-extremity complications.

## BACKGROUND

Sub-Saharan Africa is experiencing an increase in the prevalence of non-communicable diseases, including diabetes. Specifically, the prevalence of diabetes has rapidly increased over recent decades.^1^ Unmet need for diabetes care among 77% of diabetes patients^2^ results in 80% to 90% of patients with diabetes having poor glycaemic control^3,4^ and complications including diabetic foot disease with a lifetime risk of developing foot ulcer estimated at 19% to 34%.^5^ Kenya with an age-standardized prevalence of diabetes at 2.4%,^6^ has between 27.1%^7^ and 63.4%^8^ of diabetes patients having poor glycaemic control.

Diabetic foot is often a neglected chronic complication,^9^

despite being a preventable complication,^10^ with an estimated rate of foot ulcers of 4% to 61% in Africa.^11–13^ From a global perspective, complications contribute to 25% of all hospital admissions, 84% of lower limb amputations, early mortality,^14^ a huge cost burden^15,16^ and long-term detrimental effects on the quality of life of patients with diabetes.^17^ In Africa, the complication of the diabetic foot includes a 3% to 61% rate of amputation, 55% mortality rate and 0% to 77% rate of peripheral arterial disease.^11,12^ Besides, treatment of diabetic foot is also expensive costing about USD 70 annually^18^ excluding the cost of managing diabetes, which ranges between USD 528.5 to USD 684.^19,20^

Diabetic foot complications are associated with poor glycaemic control,^21^ longer diabetes duration and insulin use,^22^ combined with high blood pressure.^23^ Whilst the presence of calluses on the feet,^23^ presence of infection,^24^ and the presence of peripheral vascular disease or peripheral neuropathy in patients with diabetes increases risk.^25^ However, screening and foot care including regular inspection and examination of the at-risk foot; of patients, families and healthcare providers; appropriate footwear; and treatment of non-ulcerative pathology can help prevent amputation.^26^

Currently, various studies from low- and middle-income countries, including Nigeria, Iran and Kenya, still show poor awareness of foot care among patients with diabetes.^27,28^ For instance, in Embu and Meru Counties in Kenya, 45.1% to 51.2% of diabetes patients had poor levels of foot self-care practices which were associated with a high prevalence of diabetic foot ulcers.^29,30^ In addition, a qualitative study highlighted that delay in the presentation of diabetic foot complications is associated with a low level of knowledge and awareness of foot problems, poor health-seeking behaviours and competing for personal priorities.^31^ Importantly, even when the patient visits the hospital early, only 58% of health facilities in Kenya offer diabetes care services, of which only 74% can test blood glucose.^32^ Health education provided by healthcare providers is also biased towards blood glucose control and diet with very minimal or no messages on foot care practices.^33^

The optimal management of diabetic foot requires a multidisciplinary team approach,^34^ with the patients taking a key role in self-care. All diabetes patients are recommended to undergo an annual foot review or a three-month foot review for those with a history of diabetic foot infection.^35^ Moreover, healthcare providers should provide comprehensive diabetes education, and advise patients on their risk status to effectively support self-care practices^36^ while also screening the patients early for risk of foot ulceration.^37^ We, therefore, aimed to assess the risk of diabetic foot complications among patients with type 2 diabetes in Kenya. The findings contribute to evidence on the risk for diabetic foot among patients with diabetes and provide an overview of the state of diabetes care at a tertiary referral hospital in Kenya.

## METHODS

### Study Design and Setting

We conducted a descriptive cross-sectional study involving patients with type 2 diabetes attending the Mathari National Teaching and Referral Hospital (MNTRH) between July and October 2015 following institutional review board and ethics approval. The MNTRH is one of four national teaching and referral hospitals in Kenya offering specialised inpatient and outpatient care. It is located in Nairobi, Kenya's capital city, and runs its diabetes outpatient clinic once a week. At the time of the study, the clinic had 700 registered patients with diabetes who were reviewed regularly by a diabetes nurse and consultant endocrinologist.

### Sample Size

A sample size for a single proportion was calculated based on the estimated 12% prevalence (p) rate of diabetes in an urban setting in Kenya,^38^ with a 5% precision level (e) and 95% confidence level (z=1.96 standard deviation correspondence to 95% confidence level). Thus, the minimum sample size (n) was calculated as follows: minimum sample size (n) =z^2^p(1-p)/e^2^= 1.96)^2^(0.12)(1–0.12)/0.05^2^ = 163. The sample (nf) was adjusted for the finite study population of 700 as follows: nf = (N x n)/(N + n) = (700 x 163)/(700 + 163) = 133. The sample was adjusted by 10% for non-response resulting in 147 participants.

### Participants Selection

All the type 2 diabetes patients attending the MNTRH diabetes clinic were eligible to participate. Upon ethical approval, patients' file numbers were entered into a computer program, which generated a random sample of 147 patients. Sampled patients were invited to participate and provided with information about this study. All sampled patients consented to the study.

### Data Collection

A semi-structured questionnaire was used to collect data on participants' demographic characteristics. One trained foot care specialist performed foot examinations. This included assessing for foot ulcers, dryness, deformities, amputations, previous ulcers, calluses, and neuropathies. Peripheral sensory neuropathy was assessed using 10 monofilaments, with insensitivity at four of the 10 sites considered to indicate peripheral sensory neuropathy. Posterior tibial and dorsalis pedis artery pulses were evaluated on the same limb using a hand-held Doppler ultrasound to assess for peripheral vascular disease. Data were collected daily in English and Swahili by the researchers at the hospital's diabetes outpatient clinic until the final sampled patients were examined.

### Sample Collection Method

Urine samples for kidney function tests and blood samples for blood glucose, glycated haemoglobin (HbA1c) and lipid profile tests were collected by a trained laboratory technologist. The samples were tested within 3 hours by an accredited laboratory service provider in Kenya. Participants' blood pressure was measured on two separate occasions in a sitting position, and the average value was calculated and recorded. Weight and height were measured using a Seca® weighing scale and stadiometer, respectively, and participants' body mass index (BMI; kg/m^2^) was calculated.

### Diabetic Foot Risk Categorisation

Diabetic foot risk was categorised according to the International Working Group on the Diabetic Foot (IWGDF) consensus guidelines. The IWGDF categorises diabetic foot risk using four groups: risk category (RC) 0 = normal foot with no neuropathy; RC 1 = loss of protective sensation; RC 2 = loss of protective sensation, deformity and peripheral arterial disease; and RC 3 = previous history of ulceration or amputation.^39^ (Supplementary Table 1).

**TABLE 1:** Participants' Demographic, Clinical and Laboratory Characteristics

Demographic variables (N=147)	n	%	95% CI
Sex			
Male	44	29.9	23.0-37.9
Female	103	70.1	62.1-77.0
Age, years			
<45	20	13.6	8.9-20.2
45-54	52	35.4	28.0-43.5
55-64	47	32.0	24.9-40.0
>65	28	19.1	13.4-26.3
Marital status			
Single	15	10.2	6.2-16.3
Married	102	69.4	61.4-76.4
Widowed	18	12.2	7.8-18.7
Divorced/separated	12	8.2	4.7-13.9
Education			
No formal education	19	12.9	8.4-19.5
Primary school	58	39.5	31.8-47.7
Secondary school	58	39.5	31.8-47.7
Tertiary	12	8.2	4.7-13.9
Occupation			
Formal employee	23	15.7	10.6-22.5
Self-employed	78	53.1	44.9-61.1
Casual	12	8.2	4.7-13.9
Unemployed	34	23.1	17.0-30.7
Diabetic foot risk category*			
0 - No risk	10	6.8	2.7-10.9
1 - Low	54	36.7	28.9-44.6
2 - Moderate	53	36.1	28.2-43.9
3 - High	30	20.4	13.8-27.0
**Clinical and laboratory variables**	**Mean**	**SD**	**Range**
HbA1c, g/dL	9.2	2.2	5-15
Cholesterol, mmol/L	5.1	1.2	1.9-10
Low-density lipoprotein cholesterol, mmol/L	3.1	1.1	0.8-6.1
High-density lipoprotein cholesterol, mmol/L	1.3	0.5	0.1-4
Triglyceride, mmol/L	1.9	1.5	0-13.2
Urine albumin creatinine ratio	47.2	29.5	1-106
Fasting blood sugar	11.4	4.8	4.9-25
Body mass index, kg/m2	27.7	5.2	2-41

CI: confidence interval; HbA1c: glycated haemoglobin; SD, standard deviation.

* Risk categorisation from the IWGDF (2015), utilised in diabetic foot screening - RC 0: Normal foot with no neuropathy; RC 1: Loss of protective sensation; RC 2: Loss of protective sensation, deformity and peripheral arterial disease and RC 3: Previous history of ulceration or amputation

### Statistical Analysis

Statistical analyses were conducted using STATA version 15,^40^ with the level of statistical significance set at *p<0.05*. Descriptive statistics were used to evaluate participants' demographic, clinical and laboratory characteristics and diabetic foot risk categories. A one-way analysis of variance was performed to assess the differences between the means of diabetic foot risk categories against demographic, clinical and laboratory profiles. Post-hoc analyses were performed using Bonferroni correction to assess the differences between pairs of diabetic foot risk groups.

### Ethics

The Kenyatta National Hospital and the University of Nairobi Ethical Review Board approved this study (Ref: KNH–UON/A/303). The MNTRH hospital administration granted permission to conduct this study at the hospital. Participants provided written informed consent after all study processes and procedures had been explained to them. Serial numbers were used to ensure participants were anonymous, and the data were encrypted. Access to the data was limited to maintain privacy and confidentiality following institutional and national guidelines. Study participants benefitted from this study by receiving basic screening tests and contributing to highlighting gaps in diabetes care and management.

## RESULTS

### Participants' Characteristics

The participants were 147 patients with diabetes: 44(29.9%) men and 103(70.1%) women. The average age was 55.1 years (range 35–81 years), and the mean duration since diabetes diagnosis was 8.1 years (range 1–28 years). One hundred and five participants (59%) had HbA1c ≥7.0%, and the mean HbA1c was 9.2%(95% confidence interval [CI]: 7.7%-8.7%). The mean low-density lipoprotein cholesterol was 3.6 mmol/L (95% CI: 2.9-4.3 mmol/L)(Table 1).

### Diabetic Foot Risk Characterisation

Out of 147 participants, 30(20.4%) were at high risk (RC 3) for developing diabetic foot, 54(36.7%) were at low risk (RC 1) and 53(36.1%) were at moderate risk (RC 2). Only 10(6.8%) participants had no risk for diabetic foot (Table 1).

Participants' feet were characterised by calluses/corns (46.9%), dry and cracked skin (18.4%), oedema (13.6%) or discoloured skin (8.2%). Neurological examination revealed loss of vibration (12.2%) and peripheral sensory neuropathy (10.2%). Importantly, a vascular examination revealed a loss ofdistal posterior artery (8.2%) and posterior tibial artery (7.5%) pulses and deformity (7.5%). A minority of participants reported previous ulcers (20.4%) (Table 2).

**TABLE 2:** Diabetic Foot Clinical Characteristics

Diabetic Foot Clinical Characteristics	n	%	95% CI
Skin moist	120	81.6	75.2-88.0
Callus/corns	69	46.9	38.8-55.1
Previous ulcers	30	20.4	13.8-27.0
Dry and cracked skin	27	18.4	12.0-24.7
Ulcers	21	14.3	8.6-20.0
Oedema	20	13.6	8.0-19.2
Vibrations	18	12.2	6.9-17.6
Peripheral sensory neuropathy	15	10.2	5.3-15.2
Discoloured skin	12	8.2	3.7-12.6
Loss of distal posterior artery pulse	12	8.2	3.7-12.6
Deformity	11	7.5	3.2-11.8
Loss of posterior tibial	11	7.5	3.2-11.8

### Association between Diabetic Foot Risk and Clinical Variables for Diabetes Control

Participants in RC 2 and 3 had a high average fasting blood sugar (12.2 mmol/L and 11.3 mmol/L, respectively) and HbA1c (9.4 g% and 9.2 g%, respectively). Participants in RC 2 had the highest mean levels of serum high-density lipoprotein cholesterol (1.4 mmol/L) and urine albumin-creatinine ratio (50.3), and those in RC 3 had the lowest level of serum low-density lipoprotein (2.8 mmol/L). However, there were no significant differences between and within the means of the diabetic foot risk groups and demographic, clinical and laboratory variables (Table 3 and Supplementary Table 2).

**TABLE 3:** Differences in Diabetic Foot Risk Categories by Demographic and Clinical Variables

Variables	Diabetic Foot Risk Category, Mean (SD)				p-value^1^
	0	1	2	3	
Age, years	57 (8.89)	55.30 (11.10)	54.58 (9.82)	54.8 (9.65)	0.8674
Glycated haemoglobin, g%	9.21 (2.31)	8.90 (2.11)	9.43 (2.23)	9.21 (2.06)	0.7734
Cholesterol	5.76 (0.68)	5.15 (1.44)	5.21 (1.22)	4.74 (0.91)	0.0664
Low-density lipoprotein cholesterol, mmol/L	3.68 (0.98)	3.02 (0.98)	3.25 (1.21)	2.77 (0.85)	0.1154
High-density lipoprotein cholesterol, mmol/L	1.24 (0.36)	1.27 (0.43)	1.41 (0.63)	1.18 (0.39)	0.2142
Triglyceride, mmol/L	1.94 (1.04)	1.91 (2.00)	1.82 (1.04)	1.86 (1.02)	0.7231
Urine albumin creatinine ratio	39 (26.16)	47.04 (32.4)	50.30 (27.3)	44.7 (29.16)	0.3864
Fasting blood sugar	10.53 (4.86)	10.98 (4.92)	12.17 (4.71)	11.25 (4.87)	0.8181
Body mass index, kg/m2	29.3 (6.15)	27.46 (4.16)	27.74 (6.48)	27.76 (3.99)	0.5874

1 Differences in average clinical characteristics between diabetic foot risk was assessed using one-way analysis of variance rank test. SD, standard deviation.

## DISCUSSION

The findings demonstrate that the majority of the sampled population with diabetes had an increased risk of developing diabetic foot complications. One-fifth of participants were at high risk for developing diabetic foot, 36.7% had low risk and 36.1% had moderate risk. The proportion of patients with diabetes that had a high risk for developing diabetic foot was similar to a previous study conducted at the largest teaching and referral hospital in Kenya.^41^ However, we report higher numbers of patients with low (36.7%) and moderate (36.1%) risk for diabetic foot were higher than reported in other studies.^37,41,42^ For example, the proportion of participants with no risk of developing diabetic foot in our study was much lower than observed in comparative studies in other low-resourced countries e.g., 37.3% in Egypt,^42^ 57% in Kenyatta National Hospital,^41^ and 72.7% in Tunisia.^37^ The high proportion of patients with diabetes at high risk of developing diabetic foot in our study may be explained by several local reasons. These include a low awareness and poor knowledge of diabetes management and complications amongst patients and healthcare workers;^30,43^ inadequate or lack of proper foot care among patients with diabetes;^29,33,44,45^ and low levels of diabetic foot screening and foot self-care.^29,30,33^ The poor foot screening practice may be because tools and equipment in diabetes clinics for diabetic foot screening (e.g. Doppler ultrasound machines) are not universally available.^32^ Thus, highlighting the fact that foot care is possibly a neglected part of diabetes management. The diabetic foot risk classification has been proposed as an effective tool to prevent lower-extremity complications of diabetes and can form part of a screening system.^42,46^

The proportion of participants with previous ulcers in our study was higher than in a recent study in Kenya where only 1.6% and 3.8% of patients had a history of or active foot ulcers, respectively.^8^ This was, however, similar to previous studies in sub-Saharan Africa that reported 16%^41^ and 4% to 61%^11,12^ rates of previous foot ulceration. The high rate in our study may be explained by the high risk of developing diabetic foot among our participants, most of whom also had calluses/corns and infections that are associated with diabetic foot complications.^23,36,46^ However, in our study, we noted fewer foot deformities compared with a previous study in Tunisia where 43.6% of the participants had foot deformities.^37^

Previous studies identified several risk factors for developing diabetic foot ulceration, including longer duration of diabetes, poor glycaemic control, diastolic hypertension and poor self-care.^44,47^ Our study found that most patients with diabetes had poor glycaemic control, which increased the likelihood of developing diabetes complications and a mean duration of 8.1 years. However, similar to studies in Botswana and Saudi Arabia,^48,49^ we found no significant association between poor glycaemic control and diabetic foot risk groups; though intensive glycaemic control, which significantly decreases the risk of amputation among patients with type 2 diabetes is needed.^50^ In addition, there is a need for enhanced training of healthcare providers on comprehensive management of diabetes, health education among patients on diabetic foot prevention and management and a multidisciplinary approach to preserve limbs in low-resourced settings.^51^

Already, the Ministry of Health in Kenya through funding from the World Diabetes Foundation is investing in equipping at least 350 health centres, establishing 52 diabetic foot care centres, establishing a mobile foot care clinic for hard-to-reach areas, and training 1000 healthcare professionals at primary level and 3000 community health workers on diabetic foot care and education to strengthen prevention and management of diabetes and diabetic foot in Kenya.^52^ Moreover, at least in the capital city, evidence shows that most healthcare professionals are trained in the management of diabetes.^53^ While these efforts are being implemented, Kenya remains with a significant shortage of foot specialists resulting in untrained healthcare professionals to provide foot care, which may sometimes not be comprehensive to preserve patients' limbs.

### Strengths and Limitations

The present study was limited to one referral hospital and included a small sample; therefore, the results cannot be used to generalise the current risk of diabetic foot in Kenya. However, this study indicates the magnitude of the risk of diabetic foot in Kenya, especially considering that foot examination is not routine practice in most health facilities. Moreover, we lacked some equipment such as a Doppler ultrasound machine to measure the absence of vibratory perception (neuropathy) and a blood pressure machine to measure the ankle-brachial index. Our study did not also assess the potential confounders to developing diabetic foot including the duration of seeking care at the clinic. Despite these limitations, the study provides evidence of the possibility of using the diabetic foot risk classification system in Kenya and forms a basis for further studies on clinical outcomes after diabetic foot risk assessment.

### Implications for Practice and Health Policy

These findings highlight a need for diabetic care facilities to strengthen the provision of comprehensive diabetes care including foot examination and diabetes education. Routine management of patients with diabetes should include foot examination and risk stratification to improve the quality of care provided to this population. Healthcare providers should also undergo foot examination training to be able to screen patients with diabetes for risk of developing diabetic foot, and investment should continue to be made in foot examination tools and equipment to help detect early signs of foot ulcers. The findings also highlight that a large number of patients with mild to moderate risk of diabetic foot disease exist indicating a need for comprehensive diabetes care including foot examination and diabetes education and ongoing risk stratification to improve the quality of care provided to this population. Therefore, investment in foot examination and risk assessment training and a screening program with the availability of foot examination tools and equipment to help detect early signs of foot ulcers is essential and reduces the rate of unnecessary amputations.

## CONCLUSION

The observations in this study provide a direct assessment of diabetic foot disease and risk and foot care in Kenya among patients with diabetes receiving care at an urban referral hospital. The practice of diabetic foot screening is poor in our study setting, and efforts should be made to routinely screen patients for diabetic foot complications. Healthcare professionals, providers and patients should be sensitised about the importance of foot screening to prevent lower-extremity complications. Moreover, even in a large institution like ours, the lack of some equipment prevents accurate assessment and the extent of neuropathy and vascular supply. Notwithstanding these limitations, the study provides evidence of the high incidence of patients with moderate foot risk in Kenya and forms a basis for further studies to identify foot disease and improve clinical outcomes. As this is among the first studies in Kenya on this topic, more research is needed to explore the feasibility of diabetic foot risk stratification and the needs of this population.
